# 2775. Clinical Outcomes and Treatment-Emergent Resistance of Piperacillin-tazobactam, Cefepime, Carbapenems and Fluoroquinolones for the Treatment of Bloodstream Infections due to AmpC β-Lactamase-Producing Enterobacterales

**DOI:** 10.1093/ofid/ofad500.2386

**Published:** 2023-11-27

**Authors:** Jasanjeet Jawanda, Matthew A O’Donnell, Sunish Shah, Lloyd Clarke, Ryan K Shields

**Affiliations:** University of Pittsburgh Medical Center, Vincennes, Indiana; University of Pittsburgh Medical Center, Vincennes, Indiana; Antibiotic Management Program, UPMC Presbyterian Hospital, Pittsburgh, PA, Pittsburgh, Pennsylvania; Antibiotic Management Program, UPMC Presbyterian Hospital, Pittsburgh, PA, Pittsburgh, Pennsylvania; University of Pittsburgh, Pittsburgh, PA

## Abstract

**Background:**

Cefepime (FEP) and carbapenems (CB) are preferred over piperacillin-tazobactam (TZP) for treatment (tx) of *Enterobacterales* with significant AmpC production due to concerns for inducible resistance. We aimed to determine if dose-optimized TZP is as effective for bloodstream infections (BSI), and to establish rates of tx-emergent resistance in an era of β-lactam (BL) dose optimization.

**Methods:**

Patients with BSI due to *E. cloacae*, *K. aerogenes* and *C. freundii* from 2017 to 2022 at two hospitals were included. We excluded patients surviving < 48 hours or if polymicrobial BSI due to a Gram-negative organism resistant to BLs. Primary outcome was 30-day success, defined as survival, clinical improvement, completion of definitive tx and absence of recurrent infection. Development of resistance was assessed over 90 days.

**Results:**

429 patients were identified; 362 met inclusion criteria. The median age was 65 years, 61% were male and median Pitt Bacteremia score was 2 (**Table 1**). Median (IQR) time to *in-vitro* active tx was 7.6 (1.4-20.7) hours. 48%, 30%, and 11% of patients received TZP, FEP, and CB as empiric tx, respectively. By comparison, 15%, 47%, and 22% received TZP, FEP, and CB as definitive tx, respectively (n=344). Patients tx’d with definitive CB were more likely to receive inactive empiric tx (p=0.0004) and have complicated BSI (p=0.015). Dose optimization was more common for TZP than FEP or CB (p=0.02). Early clinical failure was more common for CB than other agents, but similar for TZP and FEP (**Table 2**). 30-day clinical success rates ranged 58-85%, were lower for patients tx’d with CB (58%; p=0.0006), and comparable for TZP and FEP (p=0.25). By empiric tx, there were no differences in early clinical failures, microbiologic relapse, 30-day success, or all-cause mortality between patients tx’d with TZP or FEP (**Table 3**). Overall, 226, 279, and 117 patients received TZP, FEP, and CB as empiric or definitive tx. Corresponding rates of resistance development within 90 days were 2%, 1%, and 3%, respectively.

Results Table 1. Patient demographics, severity of illness, infection and treatment characteristics based on definitive therapy

**Figure:**
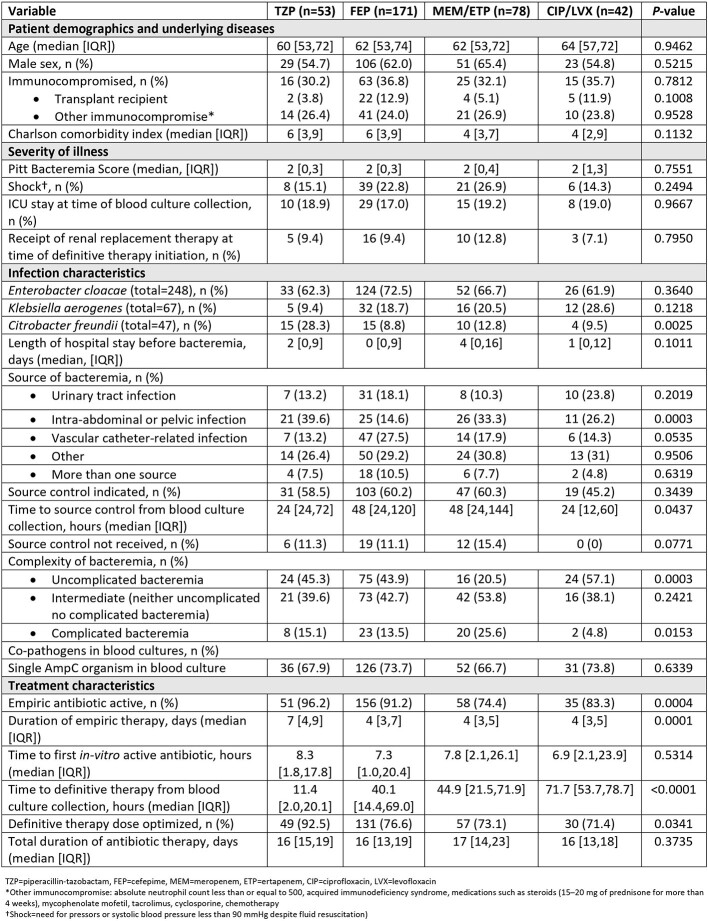

Results Table 2

**Figure:**
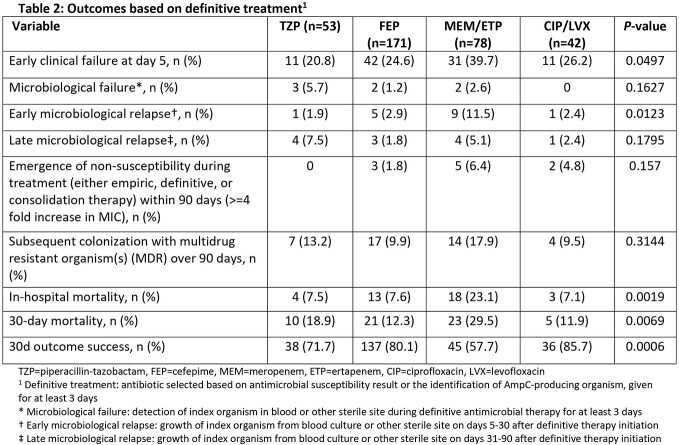

Results Table 3

**Figure:**
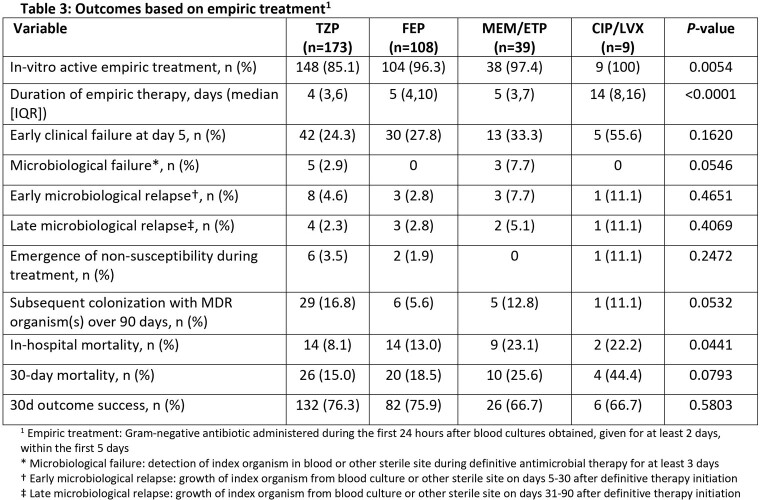

**Conclusion:**

Definitive tx with dose-optimized TZP did not lead to worse clinical or microbiologic outcomes compared to FEP, CB and FQ. There were no cases of tx-emergent resistance among patients who received TZP as definitive tx, and overall rates of resistance development were low.

**Disclosures:**

**Ryan K. Shields, PharmD, MS**, Allergan: Advisor/Consultant|Cidara: Advisor/Consultant|Entasis: Advisor/Consultant|GSK: Advisor/Consultant|Melinta: Advisor/Consultant|Melinta: Grant/Research Support|Menarini: Advisor/Consultant|Merck: Advisor/Consultant|Merck: Grant/Research Support|Pfizer: Advisor/Consultant|Roche: Grant/Research Support|Shionogi: Advisor/Consultant|Shionogi: Grant/Research Support|Utility: Advisor/Consultant|Venatorx: Advisor/Consultant|Venatorx: Grant/Research Support

